# The Krebs Cycle Connection: Reciprocal Influence Between Alternative Splicing Programs and Cell Metabolism

**DOI:** 10.3389/fonc.2018.00408

**Published:** 2018-09-26

**Authors:** Giuseppe Biamonti, Lucia Maita, Alessandra Montecucco

**Affiliations:** Istituto di Genetica Molecolare, Consiglio Nazionale delle Ricerche, Pavia, Italy

**Keywords:** splicing regulation, histone modifications, piruvate kinase, 2-oxoglutarate-dependent dioxidases (2-OGDD), lysine demethylases (KDM), m6A demethylase, DNA demethylases (TETs)

## Abstract

Alternative splicing is a pervasive mechanism that molds the transcriptome to meet cell and organism needs. However, how this layer of gene expression regulation is coordinated with other aspects of the cell metabolism is still largely undefined. Glucose is the main energy and carbon source of the cell. Not surprisingly, its metabolism is finely tuned to satisfy growth requirements and in response to nutrient availability. A number of studies have begun to unveil the connections between glucose metabolism and splicing programs. Alternative splicing modulates the ratio between M1 and M2 isoforms of pyruvate kinase in this way determining the choice between aerobic glycolysis and complete glucose oxidation in the Krebs cycle. Reciprocally, intermediates in the Krebs cycle may impact splicing programs at different levels by modulating the activity of 2-oxoglutarate-dependent oxidases. In this review we discuss the molecular mechanisms that coordinate alternative splicing programs with glucose metabolism, two aspects with profound implications in human diseases.

Cells tune their metabolic state in response to extracellular signaling and nutrient availability (1). Thus, quiescent and differentiated cells metabolize glucose through glycolysis followed by oxidative phosphorylation in mitochondria with the production of ~30 molecules of ATP for each glucose molecule (2, 3). In contrast, rapidly proliferating normal and tumor cells use energetically inefficient “aerobic glycolysis” (4) (with the production of only 2 ATP molecules) in which pyruvate, i.e. the final product of glycolysis, instead of being imported into mitochondria to fuel the Krebs cycle (also known as the tricarboxylic acid -TCA*-* cycle) is reduced to lactate and secreted in the extracellular milieu (see Figure 1). The choice between these two metabolic pathways is tightly controlled mainly by the expression and activity of pyruvate kinase M (PKM) isoforms that are altered in tumors. Although energetically inefficient, this metabolic reprogramming, known as Warburg effect, is beneficial to cancer cells since glycolysis intermediates can be used in biosynthetic anabolic pathways.

**Figure 1:**
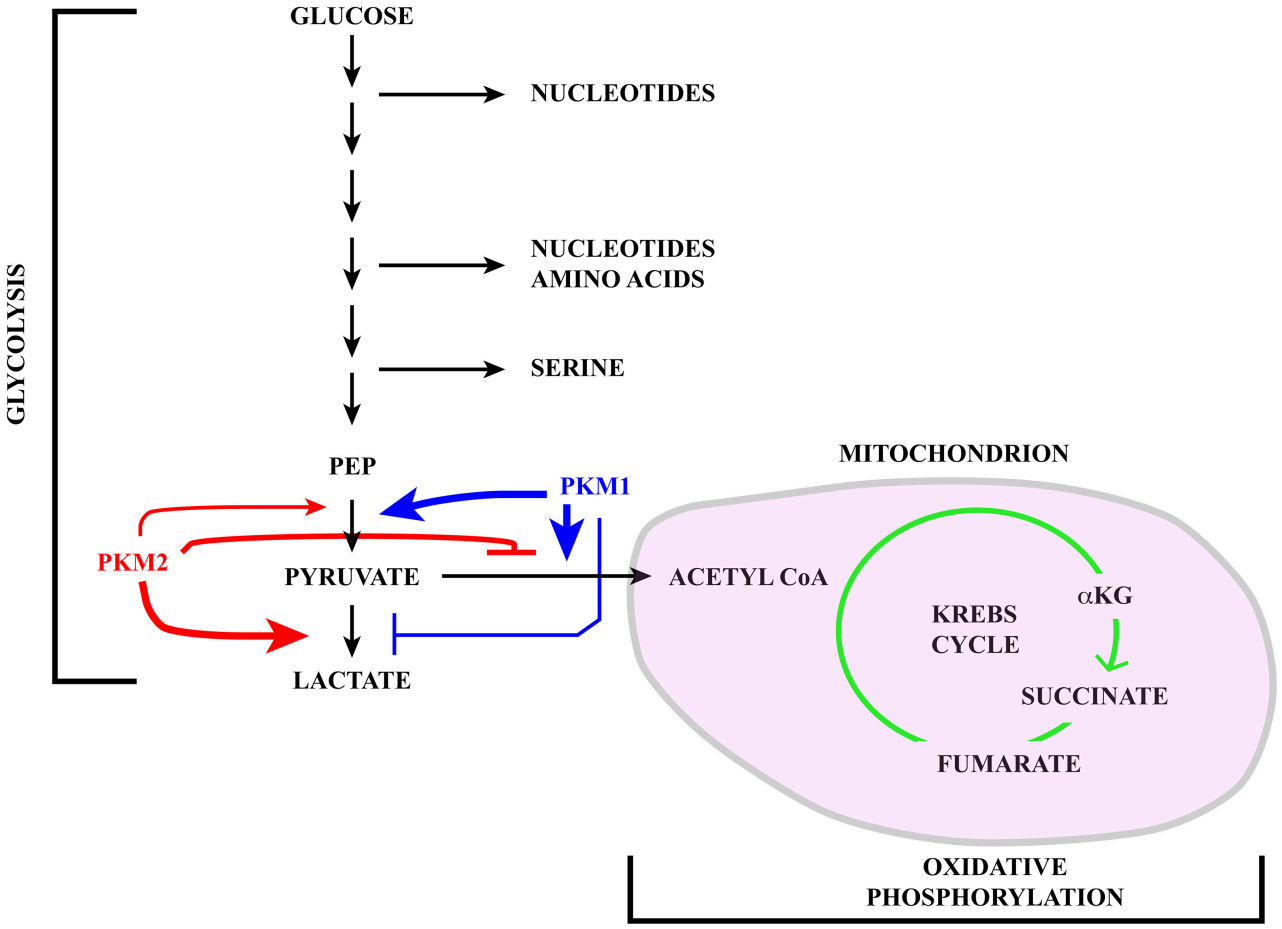
Schematic representation of the glucose catabolism highlighting glycolysis and oxidative phosphorylation in the mitochondrion (pink). Pyruvate kinase (PK) catalyzes a rate limiting step of glycolysis in which a phosphate group is transferred from phosphoenolpyruvate (PEP) to ADP, leading to the production of one molecule of ATP and pyruvate. PKM1 (blue) and PKM2 (red) are generated via alternative splicing of *PKM* transcripts. While PKM1 is constitutively active, the activity of PKM2 is regulated in response to a number of signaling pathways. Active PKM2 tetramers promote pyruvate oxidation to acetyl-CoA, in this way fueling the Krebs cycle, whereas PKM2 dimers less efficiently catalyze the last step of glycolysis, inhibit pyruvate oxidation and induce reduction of pyruvate to lactate.

Alternative splicing of gene transcripts is a potent and versatile mechanism to modulate gene expression in response to a wide range of physiological and pathological cues and stressing events. Deciphering the mechanisms that control splicing decisions and connect alternative splicing programs to the whole set of cell functions remains a main subject of investigation with profound implications for human disease. A common feature of cancer cells is the unbalanced expression of splicing variants (5, 6). This profound deregulation of splicing programs is rarely due to mutations in the affected genes or in genes for splicing factors (7). Notable exception are myelodysplastic syndromes (MDS) (8), a heterogeneous group of disorders that affect hematopoietic progenitor cells and the production of different types of blood cells, and chronic lymphocytic leukemia (9). More than 50% of MDS cases are caused by somatic heterozygous mutations in spliceosomal proteins SF3B1, SRSF2, U2AF35, U2AF65, and the U2AF-related gene ZRSR2 [for a review see (10)]. More frequently splicing deregulation originates from alterations in signaling pathways that target the expression or activity of splicing regulators (6, 11–13). Interestingly, a number of studies have recently begun to unveil the impact of cell metabolism on splicing decisions (6, 12, 14–18). Key players in this regulatory program are members of the 2-oxoglutarate-dependent dioxygenase (2-OGDD) superfamily that act as sensors of metabolic alterations and energetic stress.

In the first part of this review we briefly introduce the mechanisms underlying splicing decisions. A particular emphasis is given to the pivotal role played by chromatin organization in splice site selection, since this layer of regulation is highly sensitive to the metabolic status of the cell. Thereafter, we focus on the reciprocal influence of alternative splicing and glucose metabolism. In particular we discuss:

1. How alternative splicing of pyruvate kinase M (*PKM*) gene transcripts can impact glucose catabolism by directing the choice between glycolysis and full oxidation of glucose through the Krebs cycle;
2. The role of 2-oxoglutarate-dependent dioxygenases (2-OGDD) in controlling alternative splicing. Intermediates of the Krebs cycle influence cell features relevant to oncogenic transformation and their levels are altered by specific gene mutations (19, 20) or in response to changes in growth conditions. These oncometabolites act by modulating the activity of 2-OGDDs that specifically target DNA, RNA, and proteins.

The connection between 2-OGDDs activities and splicing programs is frequently mediated by the epigenetic landscape, which in turn is modulated in response to metabolic changes (21) by two classes of enzymes: writers and erasers. Writers include DNA methyltransferases (DNMTs), histone acetyltransferases (HATs), and histone methyltransferases (HMTs). Recently, it has been shown that epigenetic programs act also through the reversible N^6^-adenosine methylation (m^6^A) of RNA molecules by the METTL3/METTL14 complex (22). Erasers include histone deacetylases (HDACs), DNA demethylases, histone demethylases (HDMs), and m^6^A demethylases ALKBH5 and FTO.

Although the activity of most of these enzymes can be modulated in response to metabolic changes (21–23), the strongest influence of metabolism on epigenetic organization involves “erasers” of methylation patterns that belong to the large 2-OGDDs superfamily.

## Splicing regulation and chromatin

Splicing consists of the precise removal of intronic sequences from the primary gene transcript (pre-mRNA) to produce a mature mRNA molecule. This reaction is carried out by the spliceosome, a complex molecular machine assembled in a stepwise manner on the pre-mRNA and composed of five small nuclear ribonucleoparticles (U1, U2, U4, U5, and U6 snRNPs) and a large number of proteins (24). The spliceosome recognizes short elements with a loose consensus sequence at exon–intron boundaries (5′ and 3′ splice sites–5′ss and 3′ss) and at the branch point, which makes the selection of splice sites particularly challenging. Even more so if one considers that internal exons in human cells have an average size of 132 nt and are frequently flanked by introns of thousands of nucleotides in length (25). These features, on the other hand, offer the opportunity of alternative splicing (AS) events that increase transcriptome and proteome complexity (26). The vast majority (>90%) of human genes produce transcripts that undergo alternative splicing (27). AS profiles are regulated during development and cell differentiation, in a tissue specific manner and in response to physiological stimuli and different types of cell stress (28).

**Figure 2:**
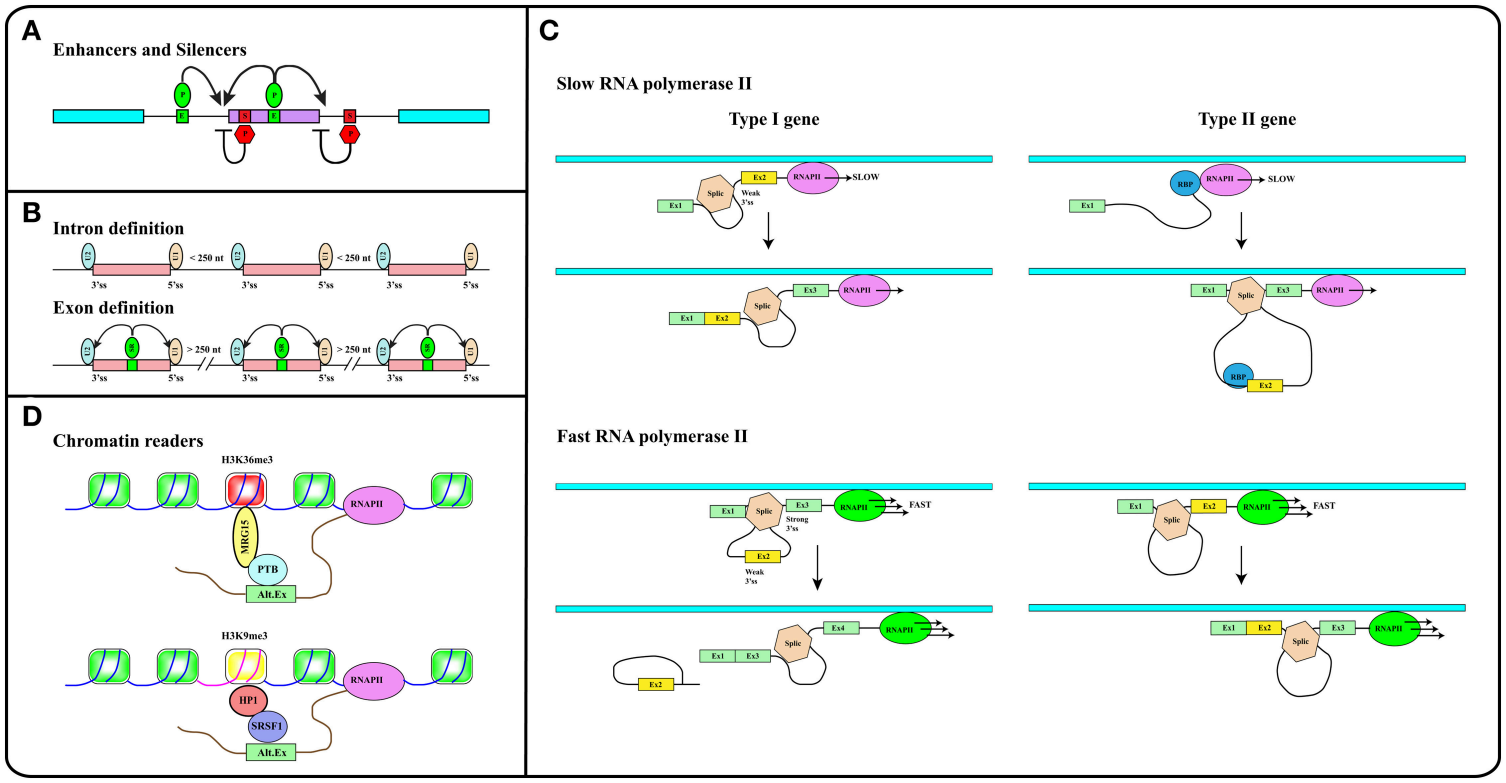
**(A)** Splicing regulatory elements, enhancers (E, green boxes) and silencers (S, red boxes) are recognized by RNA binding proteins (P) that promote (green ovals) or inhibit (red hexagons) splice sites selection. **(B)** The intron definition mode accounts for the removal of short introns while exon definition explains splicing of long introns. SR factors (SR, green oval) bound to splicing enhancers (green box) promote the recruitment of snRNP particles U1 and U2 to exon boundaries. **(C)** The elongation rate of RNA polymerase II (RNAPII) determines the pace at which relevant sequence elements emerge from the transcriptional apparatus in this way affecting splicing decisions. In Type I genes, cassette exons (yellow) are included by slow and excluded by fast RNAPII. The opposite is true in the case of Type II genes. Cassette exons in Type I genes have weaker splice sites, shorter flanking introns, and distinct sequence motifs relative to those in Type II genes. Brown hexagon: spliceosome. Blue circle: RNA Binding Protein (RBP) that blocks the recognition of the 3′ splice site upstream of the cassette exon (see Text). **(D)** Histone modifications (e.g., H3K36me3 and H3K9me3 containing nucleosomes in red and yellow respectively) are recognized and bound by readers (e.g., MRG15, HP1) that in turn recruit splicing regulators (e.g., PTB, SRSF1) to modulate recognition of alternative exons. Methylation of gene body sequences (DNA in fuchsia instead of blue) promotes methylation of histone H3 at K9 leading to HP1 recruitment to internal exonic sequences.

In addition to splice site sequences, several elements contribute to splicing decisions (29, 30). A key role is played by splicing regulatory elements (SREs), located both in exons and introns, that can promote (enhancers) or inhibit (silencers) the selection of a particular splice site (31) (see Figure 2A). SREs act by recruiting specific RNA binding proteins (RBPs). The splicing outcome, in fact, is determined by the set of RBPs bound to the pre-mRNA in ribonucleoprotein (RNP) complexes. The protein moiety of these complexes is dictated by the RNA binding specificity, concentration and post-translational modification pattern of each RBP. Two main groups of ubiquitously expressed RBPs with a role in splicing regulation have been characterized in detail: hnRNP (heterogenous nuclear ribonucleoproteins) proteins and splicing factors of the SR (Ser-Arg rich) family. However, the number of splicing regulators, some of which are expressed in a tissue-specific manner, is continuously expanding (32–34).

Splicing can follow two alternative strategies: intron or exon definition (see Figure 2B). According to the intron definition model, the assembly of the spliceosome is guided by intronic sequence elements. This strategy operates during the removal of short introns, which are the rule in lower eukaryotes (30). The excision of long introns in mammalian genes (25, 29), instead, relies on the exon definition mode of splicing, in which the exon boundaries are recognized and selected with the help of additional protein-RNA interactions established across the exon. As extensively discussed in several excellent reviews (29, 30, 35), splice site selection is influenced by two further elements: (1) the rate of transcription and (2) chromatin organization. This reflects the fact that splicing is mainly co-transcriptional (36). Studies in yeast have recently proven that the splicing reaction is usually completed as soon as the intron emerges from RNA polymerase II (RNAPII), when less than 150 nucleotides downstream of the 3′ss have been transcribed (37). Two models have been proposed to explain how co-transcriptional splicing modulates AS. According to the “recruitment model” splicing factors are recruited to the nascent RNA molecule by the transcriptional apparatus (38–40) and can tether the 5′ss of the intron to RNAPII until complete synthesis of the downstream exon (41). The “kinetic model,” in contrast, assumes that the elongation rate of RNAPII defines the portion of the transcript that is available to the spliceosome and determines the pace at which relevant sequence elements, such as splice sites and regulatory elements, emerge from the transcriptional apparatus (42). It was initially suggested that a slow RNAPII would favor inclusion of an exon flanked by weak splice sites (43) (Figure 2C, Type I genes). It is now clear that slow elongation can also favor the recruitment of inhibitory splicing factors, in this manner promoting exon skipping (44) (Figure 2C, Type II genes). Thus, the effect of the elongation rate on splicing decisions (exon inclusion vs. exon skipping) actually depends on the gene context (45). The elongation rate of RNAPII ranges between 0.5 and 4 kb/min and is influenced by exon density, CpG content and methylation pattern, and histone modifications (46) pointing to the importance of chromatin organization on AS. This is further suggested by the observation that the average exon length (132 nt) matches the nucleosome length (147 bp) (25). Genome wide approaches have shown that nucleosomes are preferentially positioned on exons with a CpG content higher than flanking introns and are rarely located over intronic regions close to splice sites (47, 48). This distribution pattern can account for the sensitivity of splicing programs to the activity of chromatin remodeling complexes, such as SWI/SNF and CDH (49, 50). It has been proposed that nucleosomes slow down the elongation rate of RNAPII and favor exon recognition by the splicing apparatus (51).

Altogether these analyses provided a completely new perspective to the field by introducing chromatin organization and the epigenetic code, namely the pattern of histone modifications, as major determinants in alternative splicing regulation (52). A large number of studies in the last 20 years have deciphered important aspects of the epigenetic code. Some histone modifications are predictive of the activity of gene promoters (53), while others, including H3K36me3 and H3K27me1/2/3, are enriched in exons compared to introns (47, 54, 55). The emerging picture is that exons are already defined at the chromatin level and that histone marks are directly implicated in the recruitment of splicing factors. This is the case of H3K4me3 that is bound by the chromatin remodeler CDH1, which in turn interacts with components of the spliceosome (50). Another example is H3K36me3 that preferentially marks constitutive exons while it is found at lower levels in alternatively spliced exons (56). This modification controls the splicing profile of numerous transcripts including the human fibroblast growth factor receptor 2 (*FGFR2*) gene transcripts, in this way affecting the Epithelial to Mesenchymal cell Transition (EMT). H3K36me3 is the landing pad for the chromodomain-containing protein MRG15 (see Figure 2D), which, in turn, recognizes splicing regulator PTB/hnRNPI (52, 57). An additional H3K36me3 “reader” is Psip1, which recruits the SR factor SRSF1 (58). Splicing factors may also directly interact with nucleosomes. Thus, splicing factors SRSF3 and SRSF1 interact with unmodified histone H3, with H3K9Ac and with H3K9Me. Interestingly, phosphorylation of histone H3 at Ser 10 during mitosis releases both splicing factors from chromatin, in this way promoting the dissociation of HP1 from chromatin (59).

As discussed in excellent reviews, also DNA methylation can influence splicing profiles (35, 60). CpG methylation is nonrandomly distributed along the genome and specifically marks exons (48). Interestingly, in transcribed genes the CpG methylation pattern correlates strongly with H3K36me3 and inversely with H3K4me2 (61). DNA methylation modulates splicing of 22% of alternative exons. Three protein factors have been identified that can translate the information contained in the DNA methylation profile of gene bodies into AS regulation: (i) CTCF, (ii) MeCP2, and (iii) HP1. CTCF and MeCP2 directly read the methylation status of DNA and control gene expression. Although mainly characterized for their activity at gene promoters, these factors also bind the gene body and determine splicing of alternative exons. Notably, DNA methylation prevents the interaction of CTCF with exonic sequences, thus relieving the inhibitory effect of this factor on the elongation rate of RNAPII. In contrast, MeCP2 recruits histone deacetylases to methylated CpG, in this manner promoting a close chromatin structure that leads to RNAPII pausing and exon inclusion. Thus, CpG methylation induces skipping of exons regulated by CTCF and inclusion of those bound by MeCP2 (35, 62, 63). The involvement of HP1 in this circuitry is certainly more complex since the three HP1 proteins do not read the methylation status of CpG but interact with histone H3K9me3. Interestingly, DNA methylation induces the H3K9 trimethylation creating a substrate for HP1 binding at the chromatin level. In turn, HP1 proteins recruit several splicing factors including SRSF3 (64) and SRSF1 (59) transferring the information from DNA methylation to splicing (Figure 2D).

## The PKM connection

The pyruvate kinase M (*PKM*) gene illustrates the impact that alternative splicing exerts on glucose metabolism. Pyruvate kinase (PK) catalyzes a rate-limiting step of glycolysis in which a phosphate group is transferred from phosphoenolpyruvate (PEP) to ADP, leading to the production of one molecule of ATP and pyruvate (see Figure 1). PKs can also influence whether pyruvate is reduced to lactate by lactate dehydrogenase (LDH) or oxidized to acetyl-CoA and CO_2_ by pyruvate dehydrogenase (PDH), in this way fueling the Krebs cycle. Mammals have two *PK* genes, *PKLR* and *PKM*, each encoding two protein isoforms. While PKL and PKR are expressed in a tissue specific manner, the choice between PKM1 and PKM2 is mainly linked to cell proliferation vs. differentiation (65).

### The impact of PKM2 on cell growth and glucose metabolism

**Figure 3:**
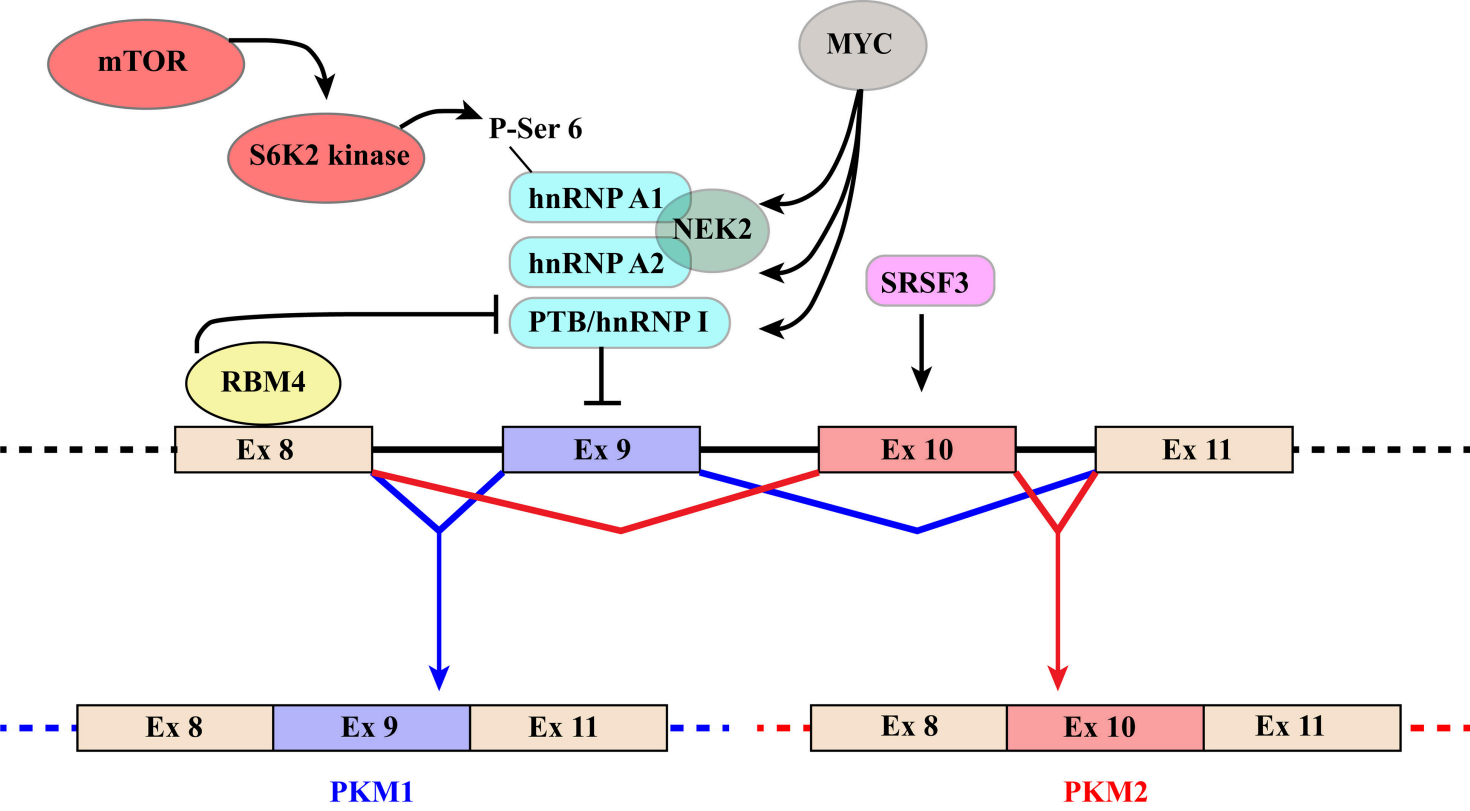
Schematic representation of regulatory mechanisms that control the switch from PKM1 to PKM2. The diagram shows the region of the *PKM* gene that contains mutually exclusive exons 9 and 10, which are included in *PKM1* and *PKM2* transcripts, respectively. Recognition of exon 9 is inhibited by hnRNPA1, hnRNPA2, and PTB/hnRNP I that bind to regulatory elements flanking the exon. Several mechanisms contribute to increase skipping of exon9 and hence PKM2 production. The MYC oncogene exerts its influence by increasing the expression of the three hnRNP proteins. NEK2 kinase, whose expression is under the positive control of MYC (66), promotes *PKM2* specific splicing through an interaction with hnRNPA1 and A2. In addition, phosphorylation of Ser6 of hnRNPA1 by S6K2 of the mTOR pathway facilitates hnRNPA1 binding to the splicing site of the PKM gene. Finally, selection of exon10 is promoted by splicing factor SRSF3. PKM1 production, in contrast, is induced by RBM4 that, upon binding to exon 8, inhibits the interaction of PTB/hnRNP I with regulatory elements controlling exon 9 splicing.

PKM1 and PKM2 are generated through alternative splicing of mutually exclusive *PKM* exons 9 and 10 (Figure 3). Even though this switch produces a modest difference of only 22 out of 531 amino acids, the effects on protein and enzymatic properties are striking. PKM1 forms stable and constitutively active tetramers and promotes the channeling of pyruvate toward the Krebs cycle in order to meet the high energy needs of normal adult tissues such as the heart, brain, and skeletal muscle (67) (Figure 1). The expression of this isoform appears to be restricted to non-proliferating cells. However, only hypotheses have been raised to explain how PKM1 favors the oxidation of pyruvate to acetyl-CoA at the expense of lactate production.

Unlike PKM1, PKM2 can promote the conversion of glucose to lactate (68), a hallmark of the Warburg effect (Figure 1). PKM2 can form either tetramers or less active dimers that differ in their affinity for PEP, with Km values 0.03 and 0.46 mM for the tetrameric and dimeric forms respectively. Although both complexes promote glycolysis, only dimeric PKM2, because of its low affinity for PEP, leads to accumulation of glycolytic intermediates, thereby providing cancer cells with substrates for anabolic processes (69). The choice between the two types of complexes is controlled by several allosteric effectors, by glucose metabolites and by post-translational modifications including phosphorylation by ERK1/2 (69–71). Moreover, PKM2, but not PKM1, can be inhibited by increased intracellular reactive oxygen species (ROS) (72) which may be produced during oxidative phosphorylation in mitochondria (73).

The impact of PKM2 on cell growth and glucose metabolism is twofold (67). As stated above, dimeric PKM2 promotes the Warburg effect, that is the shift of glucose metabolism from oxidative phosphorylation to glycolysis under normoxia (69). In addition, the PKM2 dimer is efficiently imported into the nucleus where it acts as a kinase that uses PEP as a phosphate donor to phosphorylate a number of proteins, including histone H3 at threonine (T) 11 (74). Interestingly, H3T11 phosphorylation, promotes subsequent H3K9 acetylation (74) which results in upregulation of *MYC* and *cyclin D1* genes. In turn, MYC induces the expression of enzymes connected to aerobic glycolysis (71). Moreover, nuclear PKM2 directly regulates gene transcription by acting as a coactivator of hypoxia-inducible factor-1α (HIF1α) under hypoxia (75) or β-catenin in response to EGF treatment (76) or by phosphorylating transcription factor Stat3 (77). Finally, by interacting with p53, PKM2 inhibits expression of p21 and allows proliferation of cancer cells in the presence of DNA damage (78). Altogether these properties make PKM2 more suitable than PKM1 to satisfy the anabolic requirements of rapidly proliferating cells (79). Not surprisingly, PKM2 expression is elevated in many types of cancers compared to normal tissues (65, 80, 81), including colon and breast cancers (82, 83). Moreover, it is associated with poor prognosis in signet ring cell gastric cancer and esophageal squamous cell cancer (84, 85). The switch from PKM1 to PKM2 was observed in glioblastoma and breast cancer (80, 86).

### Splicing factors involved in splicing of PKM transcripts

Because of their impact on cancer progression, the molecular mechanisms underlying the choice between *PKM* exon 9 and 10 have been investigated by several groups leading to the identification of a group of relevant splicing factors. The general strategy that directs the PKM1/2 switch relies on two main elements: (1) hnRNPA1, hnRNPA2, and PTB/hnRNPI bind to sequences flanking *PKM1*-specific exon 9 and inhibit its inclusion; (2) SR factor SRSF3 interacts with a splicing enhancer in *PKM2*-specific exon 10 and promotes its inclusion (Figure 3) (87–90). The expression of the three hnRNP proteins is controlled by MYC. This is the basis of a self-sustaining circuit in which oncogenic transformation by MYC favors aerobic glycolysis by triggering the *PKM2* specific splicing (88) and PKM2, in turn, induces the expression of MYC via histone H3 phosphorylation (71). Interestingly, PKM2 expression and glycolysis are under the control of the mTOR pathway and phosphorylation of hnRNPA1 at Ser6 by S6K2 kinase facilitates hnRNPA1 binding to *PKM* sequences (91) (Figure 3).

Additional splicing regulators have been shown to impact this general regulatory scheme. This is the case of NEK2, a serine/threonine kinase that phosphorylates splicing factor SRSF1 (92). It has been recently shown that NEK2 promotes skipping of *PKM* exon 9 and aerobic glycolysis through an interaction with hnRNPA1 and hnRNPA2 (66) (Figure 3).

Another example is RBM4, a splicing factor that links alternative splicing of *PKM* transcripts to cell differentiation programs and cancer by antagonizing the function of PTB/hnRNPI (93). RBM4 acts at different levels. Upon binding a UCUU motif in *PKM* intron 8, RBM4 lessens the interaction of PTB/hnRNPI with two UCUU motifs upstream of the 3′ splice site of the same intron, thus relieving the inhibitory effect played by PTB/hnRNPI on exon 9 inclusion (Figure 3). At the same time, it directly targets PTB/hnRNPI expression and activity by modulating the splicing profile of *PTBP1* gene transcripts. RBM4 induces skipping of *PTBP1* exon 11, which results in the production of a mRNA isoform degraded by the non-sense-mediated RNA decay (NMD) pathway. Moreover, RBM4 promotes skipping of *PTBP1* exon 9 leading to the expression of a functional PTB/hnRNPI isoform showing a reduced repressive activity on splicing. The effects on PTB/hnRNPI are consistent with the important role of RBM4 in the activation of brain-specific AS programs, in neuronal differentiation and in the switch from aerobic glycolysis to oxidative phosphorylation (93–95).

Recently, also DNA methylation has been implicated in the choice between *PKM* exon 9 and 10 (86) through the CCCTC-binding factor like protein CTCFL/BORIS, an 11-zinc-finger factor. The analysis of paired breast tumor and adjacent normal tissues has revealed a higher PKM2 mRNA and protein level in the tumor tissue, which is accompanied by an increased DNA methylation at exon 10 and a significant enrichment of BORIS at the same exon (86). Inhibition of DNA methylation impairs binding of BORIS to *PKM* exon 10 and promotes the switch from the cancer-specific PKM2 to the PKM1 isoform. Mechanistically, this involves a reduced accumulation of RNAPII at exon 10. The net effect is reversal of the Warburg effect and inhibition of breast cancer cell growth.

In contrast, high levels of PTB/hnRNPI promote proliferation, migration and invasion in clear-cell renal cell carcinoma cells *in vitro* by inducing the switch from PKM1 to PKM2. Notably, these effects are abolished upon *PKM2* knockdown (96).

## 2-OGDD superfamily

**Figure 4:**
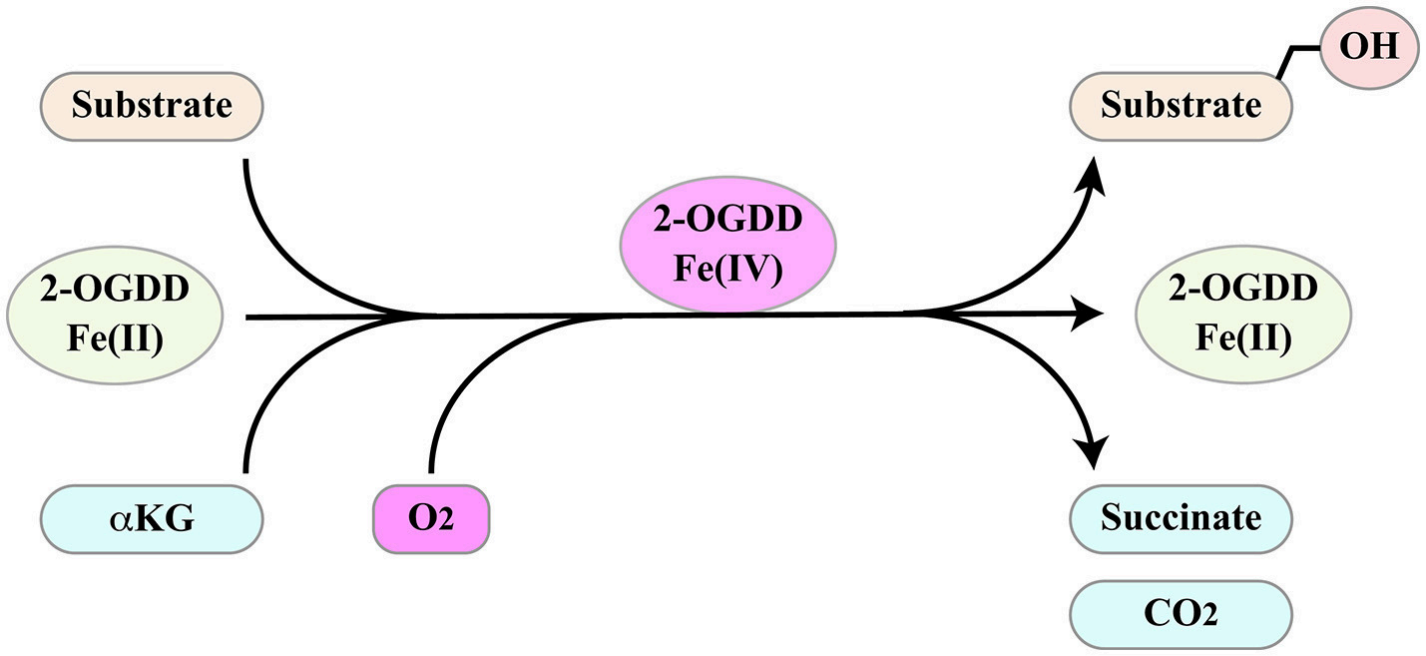
2-ODGGs catalyze the hydroxylation of a number of substrates. The reaction is coupled to oxidative decarboxylation of αKG (α-ketoglutarate/2-oxoglutarate) to succinate, two intermediates of the Krebs cycle, and depends on molecular oxygen as co-substrate and ferrous iron Fe(II) as a catalyzing cofactor.

2-oxoglutarate-dependent dioxygenases (2-OGDD) form a superfamily of enzymes with a wide range of substrates including proteins, DNA and RNA. Not surprisingly, they influence several important aspects of cell biology. Their activities and functions have been discussed in several excellent reviews (97–100). These enzymes catalyze substrate hydroxylation in a reaction that is coupled to oxidative decarboxylation of αKG (α-ketoglutarate/2-oxoglutarate) to succinate, two intermediates of the Krebs cycle (Figure 4). The reaction depends on molecular oxygen as co-substrate and ferrous iron Fe(II) as a catalyzing cofactor. Thus, 2-OGDDs can be considered metabolic “sensors,” since substrate hydroxylation is inhibited by low oxygen levels, by alterations in iron metabolism and by the αKG/succinate ratio.

The level of specific intermediates of the Krebs cycle, such as the oncometabolites succinate and fumarate and their competitor αKG is perturbed by mutations in crucial factors, with an impact on human health (101). An example is the isocitrate dehydrogenase (IDH) family that includes three members. IDHs catalyze the conversion of isocitrate to αKG but only IDH3 operates during the Krebs cycle (102). Mutated IDH1 and 2, which frequently occur in cancers such as gliomas and acute myeloid leukemia, allow the reduction of αKG to 2-hydroxyglutarate (2HG) (103). In patients bearing mutated IDH, 2HG accumulates at millimolar concentrations leading to pseudohypoxia, as revealed by HIF1α stabilization. Moreover, high levels of 2HG inhibit a number of 2-OGDDs including JmjC domain-containing histone lysine demethylases and the TET family of 5-methylcytosine hydroxylases, thus impacting epigenetic regulation (19, 104). Although the increase in CpG methylation triggered by 2HG inhibits CTCF binding to DNA (105), the effect of IDH mutants on mRNA splicing profiles has not yet been investigated.

Also inactivating mutations in fumarate hydratase (*FH*) and succinate dehydrogenase (*SDH*) genes are causative of a subset of tumors (19) and produce fumarate and succinate accumulation to millimolar levels, far in excess of physiological concentrations (106, 107). Similarly to 2HG, excess succinate and fumarate observed in *FH* and *SDH* mutants induce pseudohypoxia which stabilizes HIF1α (108) and inhibits histone demethylases and TET enzymes (109–111).

Metabolic reprogramming in cancer cells can occur independently of the mutations described above, as for instance upon upregulation of the *MYC* oncogene. In addition to promoting aerobic glycolysis, MYC can stimulate the usage of glutamine as carbon and energy source. Indeed, MYC controls the expression of genes involved in glutaminolysis, in which glutamine through anaplerotic reactions is converted to αKG and fuels the TCA cycle (112, 113). Moreover, MYC activates the expression of the tumor necrosis factor receptor-associated protein-1 *(TRAP1)* gene (114), a potent SDH inhibitor that induces succinate accumulation leading to pseudohypoxia (115).

2-OGDD enzymes share a catalytic domain that consists of a double-stranded β-helix (DSBH) core fold, frequently referred to as “jelly-roll” fold (116). Sequence comparison of the DSBH domains allowed the classification of 2-OGDDs in subfamilies (117). We mainly focus on the Jumonji-C (JmjC) domain-containing subfamily that comprises histone demethylases (KDM2-7) involved in the removal of methyl groups from specific methylated lysine (K) of histones and JMJD6 involved in splicing regulation (118). We also discuss the TET family of 2-OGDDs that target 5-methylated cytosine (5mC) and demethylate DNA (119). Finally, we consider the ALKB family, comprising FTO and ALKBH5 RNA demethylases that catalyze demethylation of adenosine (m^6^A) from nuclear RNA, which can be viewed as the RNA branch of the epigenetic program (120).

### KDMS impact splicing by modulating histone methylation

**Figure 5:**
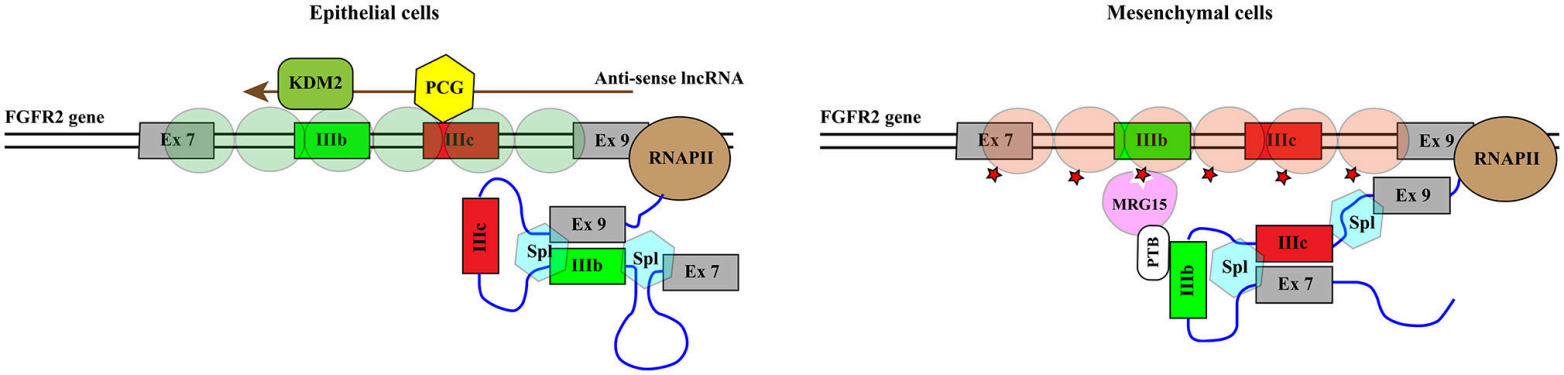
KDM2 controls the splicing profile of *FGFR2* transcripts. Schematic representation of the portion of the *FGFR2* gene comprising mutually exclusive exons IIIb (green rectangle) and IIIc (red rectangle). The azure hexagons highlight the exon boundaries selected by the spliceosome (Spl). Exon IIIb is selected in epithelial cells while exon IIIc is included in mesenchymal cells. This splicing switch is driven by the epigenetic reorganization of the region. Green circles indicate nucleosomes containing H3K27me3 and H3K4me3 while brownish red circles represent nucleosomes enriched in H3K36me3 and H3K4me1. H3K36me3 (red star) inhibits the selection of exon IIIb by recruiting the RNA binding protein PTB/hnRNPI to the nascent RNA *via* the adaptor factor MRG15. The establishment of the epigenetic landscape specific of epithelial cell depends on the expression of an antisense long non-coding RNA (lncRNA, brown arrow) generated from the human *FGFR2* locus. The lncRNA mediates the recruitment of Polycomb-group proteins (PCG, yellow hexagon), which methylate histone H3 at K27, and the histone demethylase KDM2a (KDM2, dark green rounded rectangle), which erases H3K36me3.

Histone methylation is determined by the antagonistic activity of histone methyltransferases (KMTs) and histone demethylases (KDMs) displaying different specificities in term of target residues and degree of modification, since K residues in histone tails can be mono-, di-, or tri-methylated. The histone methylation pattern has an important role in regulation of gene transcription. In general, H3K4, H3K36, and H3K79 methylation is associated with active genes, whereas H3K9, H3K27, and H4K20 methylation correlates with transcriptional repression (121). Moreover, H3K4me3 is generally enriched in promoters, whereas H3K79 and H3K36 methylations are found within the gene body. With the notable exception of the flavin-dependent monoamine oxidases KDM1/LSD1, all KDMs belong to the JmjC-subfamily of 2-OGDDs. On the basis of the sequence of their JmjC-domain, KDMs have been grouped in 7 subfamilies called KDM 2 to 8 (Table 1). All these enzymes catalyze hydroxylation of ^ε^*N*-methyl lysine leading to an unstable hemiaminal intermediate that spontaneously produces the demethylated lysine and formaldehyde (122). Each KDM displays a distinct methylation level and substrate specificity (see Table 1) (123). Moreover, KDMs show different affinities for molecular oxygen and αKG (122, 124). These features, along with the fact that some *KDM* genes are transcriptionally activated by HIF1 (125), contribute to the complex reorganization of the histone methylation pattern in response to hypoxia. Like 2-OGDDs that control HIF1α stability and activity, KDMs are inhibited by succinate and fumarate leading to an altered level of methylated histones (108). While the effect of KDMs activities on gene transcription and development is widely recognized, the impact on splicing programs is still largely unexplored. Probably the best example is provided by KDM2 that controls the splicing profile of *FGFR2* transcripts (126) (Figure 5). The *FGFR2* gene contains 2 mutually exclusive exons, IIIb and IIIc, which are differentially expressed in epithelial and mesenchymal cells. This splicing switch is driven by an epigenetic reorganization: H3K36me3 and H3K4me1 mark the alternatively spliced region in mesenchymal cells, where exon IIIc is included, while H3K27me3 and H3K4me3 are enriched in epithelial cells, where exon IIIb is selected (57). H3K36me3 inhibits selection of exon IIIb by recruiting the RNA binding protein PTB/hnRNPI to the nascent RNA via the adaptor factor MRG15. Interestingly, the establishment of the epigenetic landscape specific of epithelial cell, which impairs MRG15 binding and hence PTB/hnRNPI recruitment, relies on the expression of an antisense long non-coding RNA (lncRNA) generated from the human *FGFR2* locus. This lncRNA mediates the recruitment of Polycomb-group proteins, which methylate histone H3 at K27, and the histone demethylase KDM2a, which erases H3K36me3 (126).

**Table 1:** Human 2-OGDDs with histone lysine demethylase activity.

**GENE NAME**	**NCBI ID**	**TARGET**
KDM2A	22992	H3K36me1/2
KDM2B	84678	H3K4me3 H3K36me2
KDM3A	55818	H3K9me2
KDM3B	51780	H3K9me1/2
KDM4A	9682	H3K9me2/3 H3K36me2/3 H4.1K26me1/2/3
KDM4B	23030	H3K9me3 H3K36me3
KDM4C	23081	H3K9me2/3 H3K36me3
KDM4D	55693	H3K9me3
KDM4E	390245	H3K9me2/3
KDM5A	5927	H3K4me3
KDM5B	10765	H3K4me1/2/3
KDM5C	8242	H3K4me3
KDM5D	8284	H3K4me2/3 Male specific
KDM6A	7403	H3K27me3
KDM6B	23135	H3K27me2/3
KDM6C	7404	H3K27me3 Male specific
KDM7A	80853	H3K9me2 H3K27me2 H4K20me1
KDM7B	23133	H3K9me1/2 H4K20me1
KDM7C	5253	H3K9me1
KDM8	79831	H3K36me2
RIOX1	79697	H3K36me3
RIOX2	84864	H3K9me3

The role of RNA molecules in dictating the recruitment of KDM proteins to chromatin has been described also for KDM4D (127) adding a further layer of complexity to the system. It is plausible that lncRNAs may provide the specificity of action by bridging a specific KDM to a selected DNA sequence.

A role in splicing regulation has been suggested also for KDM5B, which targets H3K4me3. This enzyme regulates RNAPII promoter occupancy, transcriptional elongation and alternative splicing programs in embryonic stem (ES) cells. The effect on splicing decisions is partially explained by the impact of KDM5B on transcriptional elongation due to its ability to prevent H3K4me3 spreading to the gene body. In addition, KDM5B is enriched nearby alternatively spliced cassette exons and its downregulation is accompanied by altered level of H3K4me3 at alternatively spliced exons and by the differential expression of these exons (128). Interestingly, H3K4me3 nucleosomes recruit K-acetyltransferases (KATs) thus affecting the transcription elongation rate and pre-mRNA splicing of *MCL1* transcript (129).

### Bifunctional arginine demethylase and lysine hydroxylase JMJD6

The best example of a JmjC-domain-containing enzyme involved in AS regulation is JMJD6, whose structure and functions have been discussed in a recent review (16). Initially discovered as a phosphatidylserine receptor, subsequent sequence analysis identified JMJD6 as a member of the 2-OGDD superfamily (130). Several studies have implicated JMJD6 in different types of cancer such as oral squamous cell carcinoma (OSCC), colon cancer, adenocarcinoma of the lung and breast cancer (131–134). In addition, JMJD6 cooperates with MYC in reducing the p53 level in breast cancer (135).

JMJD6 is a bifunctional arginine (R) demethylase and lysine (K) hydroxylase. It has been reported to demethylate histone H3 at R2 (H3R2) and histone H4 at R3 (H4R3) (136) and to hydroxylate K residues of several proteins including p53 (132) and a subset of splicing factors (137, 138). Mass spectrometry analysis proved that U2 small nuclear ribonucleoprotein auxiliary factor 65 (U2AF65) is lysyl-5-hydroxylated by JMJD6 at positions K15, K38 and K276 (137). While the lysyl-hydroxylase function has been validated, the activity of JMJD6 in arginine demethylation is still disputed.

JMJD6 is conserved from yeast to humans. It contains a classical JmjC domain that comprises binding sites for Fe(II) and αKG, an atypical AT-hook domain, three nuclear localization signal (NLS), one nuclear export sequence (NES) and a C-terminal poly-S domain that controls the subnuclear distribution of the protein (139). A splicing isoform lacking the poly-S domain is predominantly nucleolar. In addition to interacting with a large number of proteins [for a review see 16), JMJD6 binds to single stranded RNA (ssRNA) but not to double-stranded RNA (dsRNA), ssDNA or dsDNA (140, 141). The interaction with RNA mediates the association of JMJD6 with SR factors (141) and probably involves the atypical extended AT-hook (eAT-hook) (142), in which the glycine-arginine-proline (GRP) core motif is extended with basic lysine and arginine residues in the C-terminal direction from the core but not in the N-terminal direction as in canonical eAT-hooks.

The strongest evidence in support of a role of JMJD6 in histone demethylation in living cells derives from the analysis of its interaction with the bromodomain-containing protein BRD4, a member of the bromodomain and extra-terminal domain (BET) family of proteins. The JMJD6-BRD4 complex regulates RNAPII promoter-proximal pause release by removing inhibitory factors, Hexim1 and 7SK snRNA, from the P-TEF complex. In this manner, it induces RNAPII phosphorylation and promotes the elongation phase of transcription. Critical for establishing promoter-proximal pausing is histone H4 dimethylated at R3 (H4R3me2) which is recognized and bound by the 7SK-Hexim1 complex. The JMJD6-BRD4 complex disrupts this negative regulatory element by catalyzing demethylation of H4R3me2 and decapping of 7SK snRNA resulting in the displacement of Hexim1 (143). Whether this effect on transcription elongation influences alternative splicing patterns is still to be determined. However, it is worth noticing that BRD4 is implicated in splicing decision in response to stress treatments (144) and 7SK snRNA modulates splicing by controlling transcription elongation through the P-TEF complex (145). Interestingly, splicing factors of the SR family, including SRSF2, interact with the 7SK-Hexim1 complex at gene promoters and release P-TEF and RNAPII from proximal-promoter pausing (146). It has been suggested that the recruitment of JMJD6 to gene body in a BRD4-dependent manner (147) could be important for proper RNA splicing.

Another indication of the arginine demethylation activity of JMJD6 derives from the analysis of stress granules (SG), specific cytoplasmic structures assembled in response to different types of environmental stress. It has been shown that JMJD6 is involved in SG formation by reducing mono-methylation and asymmetric dimethylation of the SG component G3BP1 at three arginine residues (148).

**Figure 6:**
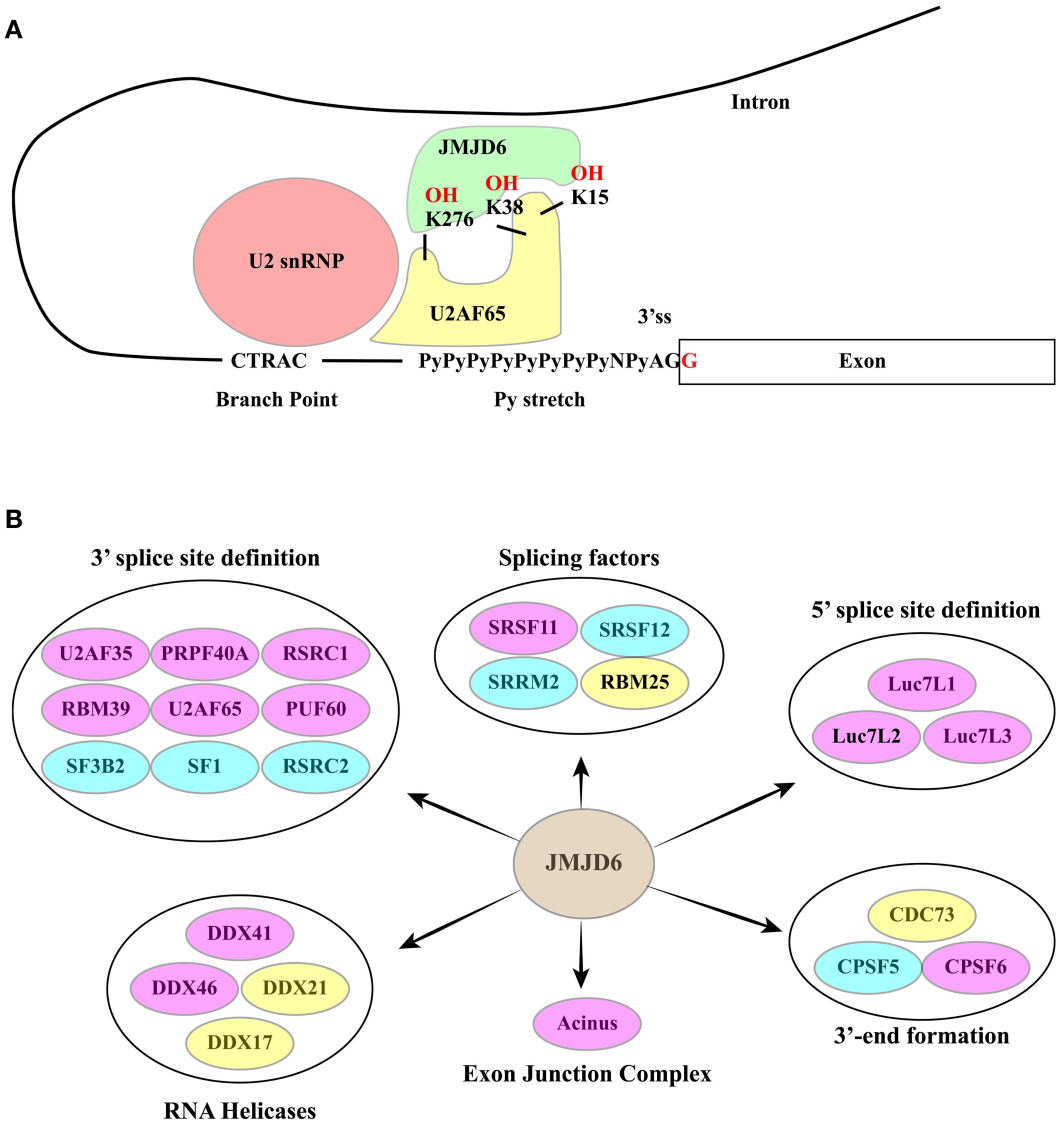
**(A)** The RNA-dependent interaction between JMJD6 and U2AF65 is crucial for the ability of the two proteins to co-regulate alternative splicing events. JMJD6 promotes the interaction of U2AF65 with the Py-stretch that assists the recruitment of U2 snRNP to the branch point. At the same time U2AF65 induces binding of JMJD6 to RNA. The RNA sequence recognized and bound by JMJD6 is still to be identified. The ability of JMJD6 to regulate alternative splicing depends on its enzymatic activity and a subset of JMJD6 / U2AF65 co-regulated splicing events require JMJD6-mediated U2AF65 hydroxylation at K15, K38 and K276. **(B)** RNA processing factors shown to interact with JMJD6. Yellow ovals: interactors described in (137). Blue ovals: interactors described in Heim et al. (141). Pink Ovals: interactors identified by both studies. Interactors are grouped according to their function in splicing. A large group of interactors is formed by proteins involved in 3′ ss definition.

JMJD6 interacts with several splicing factors, many of which contain RS domains (137, 141) (Figure 6B). However, with the exception of SRSF11, these interactors do not include “classical” SR factors (141). Notably, two of these RS domain-containing proteins, namely U2AF65 and Luc7-like 2 factor (LUC7L2), are hydroxylated at lysine residues by JMJD6 (137). The reaction is αKG- and Fe(II)-dependent and is inhibited by Krebs cycle intermediates succinate and fumarate (137). Both U2AF65 and LUC7L2 are involved in splice site selection. U2AF65 is required for U2 snRNP binding to the pre-mRNA branch point while LUC7L2 contributes to the recognition of non-consensus splice donor sites in association with U1 snRNP. In at least a subset of splicing events JMJD6 appears to act as an oxygen sensor. This is the case of vascular endothelial cell growth factor receptor 1 transcripts (VEGFR1, encoded by *FLT1*) (149). Reduced expression or activity of JMJD6 results in the production of a soluble VEGFR1 isoform which lacks the transmembrane and intracellular kinase domain and sequesters VEGF in unproductive complexes thus inhibiting angiogenesis. Critical for the effect on *FLT1* pre-mRNA splicing is the interaction of JMJD6 with U2AF65. This mechanism operates in mouse placenta (150).

A significant fraction of alternative splicing events is co-regulated by JMJD6 and U2AF65, suggesting a functional interaction between these two proteins (138). Several studies have investigated whether or not the enzymatic activity of JMJD6 is important for its function in AS regulation. However, the analysis of single genes has provided contradictory results (137, 141, 149, 151). Recently, this issue has been addressed by genome-wide approaches (138) proving that approximately 60% of splicing events regulated by JMJD6 depend on the enzyme activity. Interestingly, a subset of JMJD6 and U2AF65 co-regulated alternative splicing events are linked to JMJD6-mediated lysine hydroxylation of U2AF65 at K15, 38, and 276 and these modifications impact U2AF65 binding to RNA (Figure 6A). As expected from its role in splicing decisions, deregulation of JMJD6 function has important consequences on human health, and JMJD6 has been recently implicated in melanoma carcinogenesis through regulation of the alternative splicing of PAK1, a key MAPK signaling component (152).

### TET proteins, DNA methylation, and alternative splicing

A number of studies have proven a link between DNA methylation and alternative splicing. The CpG methylation pattern is determined by antagonistic DNA methyltransferase (DNMT1, 3A, 3B, and 3L) and demethylase ten-eleven translocations (TET1-3) activities. TET proteins catalyze the successive oxidation of 5-methylcytosine (5mC) to 5-hydroxymethylcytosine (5hmC), 5-formylcytosine (5fC) and 5-carboxylcytosine (5caC) (153, 154), which are then rapidly removed by the action of thymine-DNA glycosylase (TDG) (153, 155). While a comparable level of 5mC is detectable in all tissues (approximately 5% of all Cs), oxidation products are significantly less abundant and their levels appear to be tissue specific. Thus, 5hmC peaks in the central nervous system (<0.07%) while the abundance of 5fC and 5caC is at most 10- to 100-fold lower [for a review see 156). In the last decade 5hmC has been shown to play a direct role in gene expression regulation by modulating the activity of promoters and transcriptional enhancers (157–159). More recently, this modification has been also proven to modulate alternative splicing (62, 160, 161). Indeed, DNA methylation is enriched in cassette exons that are included in the mature mRNA, while it is under-represented in skipped exons (62), in introns, intronless genes, and pseudoexons (60). The hypothesis that methylation “marks” exonic DNA for successive recognition of the nascent transcript by the spliceosome has been recently validated by the Oberdoerffer's group that identified the underlying molecular mechanism (161). Using the *CD45* gene as model system, the authors showed that alternative splicing of a *CD45* cassette exon is controlled by the methyl-sensitive zinc-finger protein CCCTC- binding factor (CTCF) whose interaction with DNA promotes RNAPII pausing and exon inclusion in naïve peripheral lymphocytes. The increased level of CpG methylation in the *CD45* gene body, which occurs in activated lymphocytes, hampers CTCF binding and results in exon skipping. Crucial to this regulation, is the activity of TET1 and TET2 that declines during lymphocyte activation. These two factors catalyze 5mC oxidation to 5hmC and 5caC thus creating the binding site for CTCF. It is known, in fact, that CTCF is a specific reader (162) that protects 5caC from the TDG activity (153). Interestingly, TDG downregulation increases the level of intragenic 5caC and reduces RNAPII elongation (163). Thus, TET-catalyzed oxidation of 5mC appears to control alternative splicing events in a CTCF-dependent manner. It is plausible that additional readers of 5mC, or of the oxidized products generated by TET enzymes, may contribute to alternative splicing decisions (164). An example is MeCP2 that upon binding to 5mC modulates the alternative splicing programs of the BDNF transcripts (160).

TET enzymes belong to the large 2-OGDD superfamily (119, 165) and their activity is inhibited by succinate, fumarate, and hydroxyglutarate that accumulate in cell lines with mutated enzymes of the Krebs cycle [for a review see 100). For example, 5hmC is absent in nearly all gastrointestinal stromal tumors (GISTs) bearing a mutated succinate dehydrogenase SDH complex which leads to succinate accumulation (166). Thus, TETs act as sensors that modify the epigenetic organization in response to changes in the metabolic status of the cell. Intriguingly, these enzymes are induced by hypoxia, namely a condition that inhibits 2-OGDD activities. This paradox is explained by the fact that TET enzymes, thanks to their low Km for O_2_, can work even under low oxygen tension (109).

*TET2* is mutated in several human cancers, including myeloid malignancies such as acute myeloid leukemia (AML) (167, 168, 169). In particular, AML frequently shows mutations that increase the Km of TETs for αKG or produce higher level of fumarate and succinate (109). All these conditions inhibit TET activities and increase the level of 5mC. Interestingly, genomic analysis of AML patients revealed frequent mutations in genes involved in epigenetic regulation (*TET2, TET1, DNMT3A*, and *DNMT1*) as well as mutations in splicing factor SFPQ and in the non-classic regulator of mRNA processing CTCF (170). A plausible hypothesis is that the disease involves a direct (mutation of splicing factors) or indirect (CpG methylation pattern) perturbation of splicing programs. A similar association can be envisaged in myelodysplastic syndromes (MDS), a highly heterogenous group of hematopoietic tumors. The most common mutations detected in MDS patients occur in genes for RNA splicing (*SF3B1, SRSF2, U2F1, ZRSR2*) and DNA methylation factors (*TET2, DNMT3A, IDH1/IDH2*) (171, 172). This link between epigenetic and splicing factors is also suggested by the analysis of mastocytosis, a rare and chronic disease frequently caused by mutations in TET2 and SRSF2 genes (173, 174).

### m^6^A: signaling for mRNA splicing

Finally, we would like to briefly discuss the fact that specific 2-OGDDs may impact splicing by directly targeting gene transcripts. RNA molecules undergo a large number of chemical modifications. The most prevalent and also the only one shown to be reversible is methylation of adenosine at position N6 (m^6^A). As in the case of DNA and histone modifications that determine the epigenetic organization of chromatin, writers, readers and erasers of m^6^A have been identified, leading to the concept of epi-transcriptome (175). These studies also led to identify a loose consensus sequence (RRACH R = purine and H = A, C, or U) associated to this modification. In spite of this degeneracy, however, m^6^A is particularly enriched in the 3′ untranslated regions (3′UTRs) and within internal exons (176). This modification is established by the METTL3 - METTL14 heterodimer (177) and erased by FTO (fat mass and obesity-associated) and ALKBH5, two demethylases belonging to the 2-OGDD superfamily (178–180). Several m^6^A readers have been identified, including nuclear YTHDC1/2 (181), cytoplasmic YTHDF1/2/3 (181) and nuclear hnRNPA2/B1 (182). The interaction between hnRNPA2/B1 and m^6^A influences the splicing profile of target RNAs (182) while YTHDC1 affects splicing decisions by recruiting splicing factors of the SR family (183). A direct involvement of FTO and ALKBH5 demethylases in splicing has been demonstrated (179, 184– 186). Studies in mice suggest a link between FTO, obesity and metabolic syndrome by driving obesity-prone behaviors (187). Moreover, FTO-mediated m^6^A demethylation controls splicing of adipogenic regulatory factor RUNX1T1 (184). However, both FTO and ALKBH5 are unlikely to work as αKG sensors since the Km value of 2.88 μM is up to 10-fold lower than the estimated intracellular concentration of this intermediate of the Krebs cycle (188).

## Concluding remarks and therapeutic perspectives

Alterations in metabolism enable cancer cells to sustain their high proliferation rate. Oncoproteins and tumor suppressors have a major role in metabolic reprogramming (20, 189) and their unbalanced expression or mutation directly impacts the cell import of glucose and amino acids and the biosynthesis of macromolecules (190). Thus, both MYC and RAS promote glucose uptake and its utilization in aerobic glycolysis. However, they follow different strategies: RAS induces PKM2 phosphorylation by ERK resulting in the assembly of inefficient PKM2 dimers that shift glucose metabolism to glycolysis (191); MYC, in contrast, controls splicing of PKM transcripts promoting the switch from PKM1 to PKM2. In addition, it enhances the utilization of glutamine to fuel the Krebs cycle, a phenomenon known as glutamine “addiction” of cancer cells (112). Targeting the mechanisms underlying alterations of cell metabolism induced by an oncoprotein, therefore, can be a promising therapeutic strategy to treat tumors (192). Notably, MYC controls the expression of numerous proteins involved in RNA metabolism and splicing [for a review see 193), which accounts for the sensitivity of MYC-driven tumors to pharmacological inhibition of splicing (194). This feature opens the possibility to use splicing factor effectors of MYC as target for therapeutic approaches (193, 195). Since hnRNPA1, hnRNPA2, PTB/hnRNPI are crucial for the MYC ability to modulate the PKM2/PKM1 ratio and cell metabolism, manipulation of their expression levels may be exploited for cancer therapy (193, 196, 197). An alternative more promising strategy to manipulate the PKM2/PKM1 ratio is based on Anti Sense Oligonucleotides (ASO) designed to block the enhancer sequence in PKM exon 10 (198). Although the efficacy of this method in reducing PKM2 expression has been proven only in cultured cells, ASO have been successfully exploited to treat SMA (199, 200), a genetic disorder of the central nervous system. Thus, it is plausible that ASO may be tested also for treatment of glioblastoma and breast cancer in which the PKM1/PKM2 switch has been detected (80, 86).

In addition to being effectors of oncogenic pathways in controlling cell metabolism, splicing factors are targets of metabolic stress. Tumors are characterized by a significant microenvironmental heterogeneity, with some cells being in oxygen-rich districts and others distal to the blood vessels. Low oxygen tension activates HIF1. MYC collaborates with HIF1 to attenuate mitochondrial respiration and to increase glycolysis for adaptation to the tumor microenvironment. Moreover, HIF1 itself is able to orchestrate the reorganization of gene expression and splicing programs (17, 201). HIF1 increases the expression of CLK1, a kinase that specifically phosphorylates and modulates the activity of SR splicing factors (202). Thus, compounds able to modulate the activity of splicing kinases or splicing factors can be considered as possible approaches for inhibiting the response to hypoxia (195, 203, 204) in tumors.

HIF1 activation is also induced by increased levels of oncometabolites (succinate, fumarate, 2HG) which are generated by the Krebs cycle. This occurs in a subset of tumors due to mutations in specific metabolic enzymes. Mutated isocitrate dehydrogenase 1, which produces the accumulation of D-2-hydroxyglutarate (2HG), prevents the differentiation of erythroleukemia cells by affecting TET proteins, while mutations in succinate dehydrogenase (SDH) and fumarate hydratase (FH) are found in paragangliomas and pheochromocytomas, and leiomyomas and renal cell cancer, respectively [for a review see (19)]. Cell treatment with αKG can counteract the effect of oncometabolites and reverse pseudohypoxia with anti-tumor and anti-angiogenic effects (205) although its impact on splicing profiles is still to be investigated.

The main question concerns how the metabolic status of the cells is detected and how sensors may modulate splicing decisions. Studies in the last 20 years have shown that members of the 2-OGDD superfamily have a role in this regulatory circuit and that their activity is modulated by intermediates of the Krebs cycle. These enzymes have the potential to impact splicing by targeting chromatin organization, components of the splicing machinery and the RNA molecule. However, their contribution to splicing programs is still largely unexplored, particularly in the case of KDM proteins that modulate the histone code. Thus, for instance, the mechanisms conferring specificity to KDM proteins are still to be deciphered. It is likely that long non-coding RNA may have a role in this phenomenon (126), adding a further level of complexity in this regulatory circuit. Moreover, very few transcriptomic analyses have been performed so far to investigate the impact of 2-OGDDs on splicing decision. Because of the relevance to several important human diseases, such as diabetes, cancer and neurological disorders, the dissection of the link between glucose metabolism and alternative splicing in the next few years is conceivable to open new perspectives of therapeutic intervention.

## Author contributions

GB and AM designed and wrote the manuscript. All the authors revised and approved the manuscript.

### Conflict of interest statement

The authors declare that the research was conducted in the absence of any commercial or financial relationships that could be construed as a potential conflict of interest. The reviewer TLS and handling Editor declared their shared affiliation.
